# Quantitative phase contrast imaging of a shock-wave with a laser-plasma based X-ray source

**DOI:** 10.1038/s41598-019-55074-1

**Published:** 2019-12-11

**Authors:** F. Barbato, S. Atzeni, D. Batani, D. Bleiner, G. Boutoux, C. Brabetz, P. Bradford, D. Mancelli, P. Neumayer, A. Schiavi, J. Trela, L. Volpe, G. Zeraouli, N. Woolsey, L. Antonelli

**Affiliations:** 1 Empa, Materials Science and Technology, 8600 Dübendorf, Switzerland; 2 Dipartimento SBAI, Università di Roma “La Sapienza”, 00161 Rome, Italy; 3 Universitè de Bordeaux, CNRS, CEA, CELIA, UMR 5107, F-33405 Talence, France; 4 GSI Helmholtzzentrum für Schwerionenforschung GmbH, 64291 Darmstadt, Germany; 5 Donostia International Physics Center (DIPC), 20018 Donostia, Spain; 6 CLPU, Centro de Laseres Pulsados, Building M5, 37185 Villamayor, Salamanca Spain; 7 Universidad de Salamanca, Patio de Escuelas 1, 37008 Salamanca, Spain; 8 Department of Physics, York Plasma Institute, University of York, York, YO10 5DD United Kingdom; 9 National Research Nuclear University MEPhI, Department of Plasma Physics, 115409 Moscow, Russia

**Keywords:** Laser-produced plasmas, Imaging techniques

## Abstract

X-ray phase contrast imaging (XPCI) is more sensitive to density variations than X-ray absorption radiography, which is a crucial advantage when imaging weakly-absorbing, low-Z materials, or steep density gradients in matter under extreme conditions. Here, we describe the application of a polychromatic X-ray laser-plasma source (duration ~0.5 ps, photon energy >1 keV) to the study of a laser-driven shock travelling in plastic material. The XPCI technique allows for a clear identification of the shock front as well as of small-scale features present during the interaction. Quantitative analysis of the compressed object is achieved using a density map reconstructed from the experimental data.

## Introduction

Advanced X-ray diagnostics can benefit many areas of physics. These include the study of matter in extreme states, inertial fusion (ICF)^1,2^, medical physics and biophysics^3–5^ etc. For instance, within the domain of high energy density physics, time-resolved X-ray radiography has been used to follow the dynamics of a laser-driven shock-wave travelling in a target material^6–8^. The nanosecond evolution of the shock-wave requires an X-ray pulse in the picoseconds regime and high photon flux to freeze the phenomenon in a single-shot experiment. The standard technique employed to observe shock-waves is based on the absorption of photons travelling through matter^6–8^. However absorption X-ray radiography has several drawbacks. In particular, it is well known that strong shocks do not allow compressing matter more than a factor of about 4. In many cases, this is not enough to ensure sufficient contrast. Furthermore, when thick or complex targets are used (as in many ICF-related experiments^1,2^) one must use quite hard X-rays and again this leads to poor X-ray absorption contrast. These drawbacks might be overcome by using X-ray Phase Contrast Imaging (XPCI^9^). This technique relies upon the phase shift induced by an object on the travelling e.m. waves. Being particularly sensitive to density gradients it allows to probe (poorly absorbing) low-Z targets, as well as to observe subtle features which are invisible with absorption radiography^10^. In particular, XPCI results in contrast enhancement at the interfaces and it is therefore particularly suited to the study of shock-waves, as demonstrated in recent works^11–21^.

**Figure 1:**
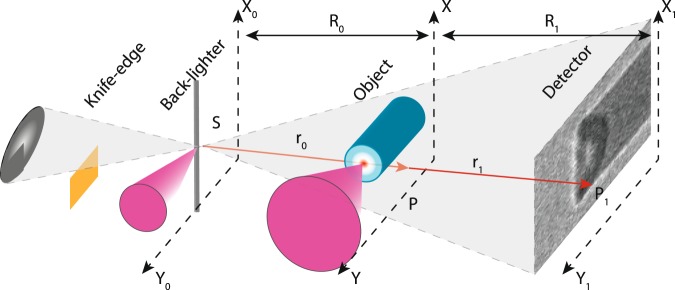
Experimental configuration: A knife edge for source characterisation is positioned to the left of the backlighter and the XPCI imaging system is positioned to the right.

In this work, we present the analysis of a laser-driven shock-wave via XPCI. In particular the XPCI simulation code, already employed by *Antonelli et al*.^18^ is now explained in details. Moreover a different approach is followed. A density map is reconstructed from the measured image by combining a phase-retrieval code coupled with a tomographic reconstruction algorithm. The experiment was performed at the Petawatt High-Energy Laser for Heavy Ion EXperiments (PHELIX) in GSI (Germany)^22^, employing the configuration shown in Fig. 1. We used two laser pulses: a long pulse (nanosecond time duration) to launch the shock and a short pulse (half picosecond time duration) to generate the X-ray source. We employed a propagation-based XPCI geometry that does not require optics (crystals) or interferometers (based on transmission gratings)^23,24^, making it suitable for use on large laser fusion facilities like NIF^25^ and LMJ^26^. In propagation-based XPCI experiments, X-rays propagate freely in the vacuum after they have passed through the object of interest. The distance between the object and the detector determines the degree of interference between phase-shifted and unperturbed rays.

Our X-ray source was produced by irradiating a solid target at intensities larger than 10^17^ W/cm^2^, generating hot electrons that cross the backlighter target. These hot electrons can interact with bound electrons in the atoms, generating vacancies that are immediately filled by electrons from higher shells. The photons that are emitted during this bound-bound transition have wavelengths dependent on the transition energy e.g. L-shell to K-shell transitions result in *Kα* emission. These sources are characterized by subpicosecond time duration^27^ (shorter than the hydrodynamic time) and a spectrum with few energetic lines (*Kα*, *Kβ* etc.) sitting on Bremsstrahlung continuum^28^. The energy conversion efficiency, from laser to X-rays, for on-target laser intensities above 10^18^ W/cm^2^ oscillates between^29^ 1 × 10^−5^ and 3 × 10^−4^. As an example, using 1 J laser pulse, the X-ray photon flux for the copper *Kα* transition (8.047 keV) is 8 × 10^10^ photons/4*π* sr/pulse. These characteristics make laser-driven X-ray sources attractive candidates for X-ray imaging in experiments at high-power laser facilities.

In our experiment, we used a 5 m diameter tungsten wire as a backlighter. Using a wire instead of a flat foil limits the spread of electrons in the target and hence limits the source size^29^. Tungsten wire was used in previous experiment at this facility^30^, providing good performance in terms of photon flux, energy and, above all, source size. A small source size makes XPCI possible in such a configuration. Tungsten can be manufactured easily down to 5 m diameter wire. Mass limited target results in a better flux compared to pin-holes which limit the flux. The wire was illuminated by a laser pulse with the following parameters: 0.5 ps time duration, maximum pulse energy of 25 J and wavelength of 1.06 m. The focusing optics used for the backlighter pulse was a 45 deg f = 400 mm off-axis parabola. The FWHM of the focal spot was ~5 m, the energy encircled in this diameter is ~6 J (1/4 of the pulse energy), leading to on-target intensities of around 6 × 10^19^ *W*/*cm*^2^ for the backlighter beam.

The X-ray source was used to image a shock launched inside a polystyrene cylinder (300 m diameter and 300 m long) using a second laser pulse with a wavelength of 1.06 m, energy of 25 J and adjustable time duration between 1 and 10 ns. The focusing optic was a 90 deg f = 1500 mm off-axis parabola leading to a focal spot diameter of 50 m, which corresponds to an intensity on the target of 3 × 10^14^ *W*/*cm*^2^. We did not use any smoothing techniques (random phase plate etc.). The interaction face of the plastic cylinder was coated with a 100 nm-thick layer of aluminium in order to increase laser absorption and avoid laser shine-through at early times. The time delay between the two pulses was varied between 1 and 10 ns. We chose a plastic object for two reasons. First, the transmission of polystyrene in the X-ray photon range (6–9 keV) is above 70%, allowing for a clear demonstration of the performance of XPCI. Second, we have checked that the EOS used by DUED reproduces with high accuracy the experimental data^31^ for plastic at pressures in the range 1–10 Mbars.

## Result

A real image is given by the convolution of several contributions, summarized by the following formula^32–34^:

1 $${I}_{real}={I}_{ideal}\ast PS{F}_{{\det }}\ast {\sigma }_{s}[{R}_{1}/{R}_{0}]$$

where *I*_*real*_ is the “real” observed image, *I*_*ideal*_ the image obtained assuming an ideal point-like source and perfect detector, *PSF*_*det*_ is the detector point spread function (PSF), and *σ*_*s*_ is the source size. The last parameter has to be rescaled by the ratio *R*_1_/*R*_0_ (where *R*_0_ is the source-object distance and *R*_1_ is the object-detector distance). The blurring effect induced by the source is proportional to *R*_1_, meaning that the smaller the value of *R*_1_, the lower the negative effects due to the finite source size.

To help to choose the correct distances, we developed a simulation code based on the Fresnel-Kirchoff formalism^35^ (cf. Image Simulation in Methods), allowing generating a synthetic XPC-image starting from a density map generated by hydrodynamic codes (in particular we used the code *DUED*^36^). The parameters used to simulate the shock are the same as those detailed above (laser and object), while the X-ray source was modelled as a Gaussian beam with a FWHM of 5 m and 7.5 keV photon energy. The X-ray energy is close to the average one for a tungsten Bremsstrahlung backlighter.

**Figure 2:**
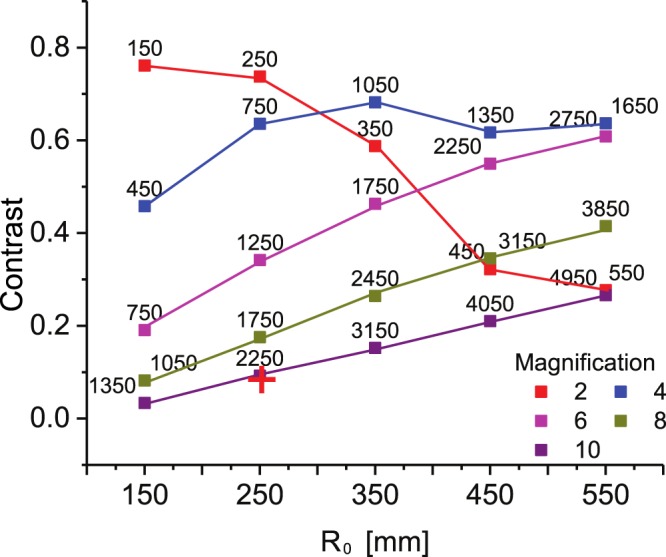
Calculated Contrast plotted against the source-object distance *R*_0_ for a simulated shock-wave at different magnifications. The numbers on the top of the dots are the corresponding *R*_1_ values. The configuration used in the experiment is represented by the red cross.

Figure 2 shows a preliminary study of the simulated shock *Contrast*^37^ versus *R*_0_ at different magnifications. For the evaluation of *Contrast* = (*I*_*max*_ − *I*_*min*_)/(*I*_*max*_ + *I*_*min*_), the maximum (*I*_*max*_) and minimum (*I*_*min*_) light intensity in the neighbourhood of the shock-front were considered. From the plot in Fig. 2, it is clear that increasing the *Contrast* at magnifications higher than 4 requires the distance *R*_0_ to be increased as well. This behaviour can be explained by the sensitivity of the propagation-based geometry to source lateral coherence *L*_⊥_ = *λR*_0_/*σ*_*s*_, where *λ* and *σ*_*s*_ are the source wavelength and size respectively. The spatial coherence requirements for propagation-based XPCI have been studied by Wu *et al*.^38–40^. Our experimental configuration is represented in Fig. 2 by a red cross, where *R*_0_ = 25 cm and *R*_1_ = 205 cm. This operating point was chosen as a compromise between the geometrical constraints imposed by the vacuum chamber, the layout of the laser optics and the detector resolution. Our detector was an SR-type imaging plate (IP-SR), with 100 m intrinsic resolution^41,42^. The IP was installed in air, after a 0.5 mm-thick PMMA window and filtered with 13 μm-thick aluminium foil ensuring that all X-ray photons with energies below 5 keV are suppressed.

**Figure 3:**
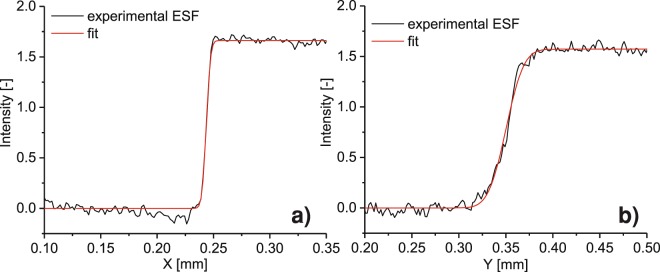
Source Edge Spread Function (ESF) along the X-axis (**a**) and Y-axis (**b**); measured ESF (black), and the fit curve (red) are shown.

On each shot, the backlighter spectrum was monitored with a HOPG crystal spectrometer^43^ and a gold knife edge was used to measure the source size. The knife edge (see Fig. 1) was placed 55 mm from the source and 152 cm from the detector (IP-SR). The high (26x) magnification used for the *ESF* ensured that the source-extension contribution dominated over the detector *PSF*. In Fig. 3, the source *Edge Spread Functions* (*ESF*) along the *X* and *Y* axes are shown for a single shot. The experimental curve is fitted with a function obtained by convolving a Gaussian with a Heaviside step function. The source widths are different along the two axes, due to the geometry used to illuminate the tungsten wire and the processes involved in laser-matter interaction. As observed by Park *et al*.^29^, the source size is bigger than the laser focal spot due to lateral spreading of hot electrons in cold matter. In our case, the width along the X-axis was constrained by the wire diameter (5 um), which means that the resolution on this axis is limited by the intrinsic detector resolution, corresponding to 10.6 um on the object plane. On the Y-axis, no physical constraints were present besides the wire length. Hence, due to an average source size of 30 um, the resolution was limited to 27 um. The average photon energy was measured to be around 7.5 keV. The source size so measured is later used to simulate the XPC-image, after being rescaled by the source magnification (*R*_1_/*R*_0_) as reported in Eq. 1.

In order to compare experimental and measured images, both are normalized using a flat-field image without the object. During the experiment, it was not possible to simultaneously acquire an object image and a flat-field image. Moreover, the reproducibility of the X-ray source (in terms of flux, source size and spectrum) was not sufficient to allow using a single flat-field measurement for all data. Therefore starting from the acquired image, we estimated the flat-field performing a polynomial fit to each column within the image, excluding any pixels containing the object.

**Figure 4:**
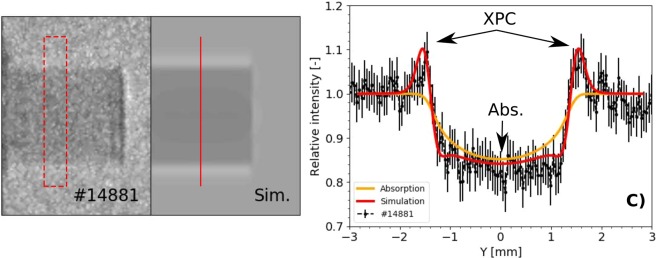
Left: Measured image without filter. The experimental profile shown in (**c**) was constructed from data inside the red-dashed box. Centre: Simulated image taking into account phase-contrast and absorption. The red vertical line marks where the simulated profile in (**c**) has been sourced from. Right: A superposition of the two intensity profiles. The experimental values are represented in black, while the red line is taken from the simulation (XPCI + absorption). The absorption effect (*Abs*.) and the phase-contrast one (*XPC*) are clearly identifiable.

XPC-images of the object without a laser-driven shock are shown in Fig. 4. Experimental images are on the left-hand side (shot #14881) and the simulated versions are positioned on the right (labelled as *Sim*.). The synthetic image was generated using a density map of the unperturbed object, the measured spectrum and source size (5 × 30 μm) as source parameters. Profiles taken along the object axis for both images are shown in Fig. 4c. In order to improve the signal-to-noise ratio we performed an average over a 10 pixel width area (shown in red in Fig. 4). The agreement between the two profiles, confirms that the code is able to reproduce both phase-contrast and absorption. The first one is clearly visible as contrast enhancement at object boundary. Pure absorption contribution is visible in the middle of the object.

**Figure 5:**
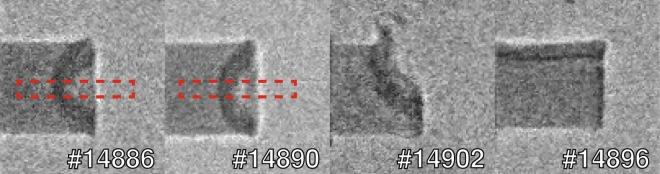
Phase contrast images of shock-waves. The object diameter is 300 m and the laser entered from the right in each figure. The red boxes inside images #14886 and #14890 circumscribe the area that was averaged over to generate the profiles.

**Figure 6:**
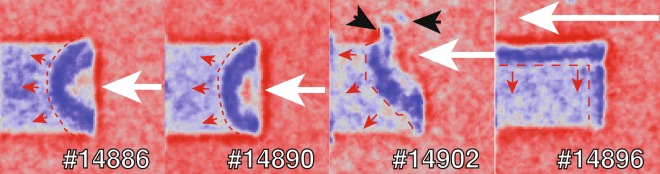
Filtered images from Fig. 5 with enhanced contrast. The intensity ranges from 0 (red) to negative values (blue). The white arrow represents the laser, the red line and arrows indicate the shock front and expansion direction, while black arrows indicate pieces of plastic blown off during the interaction.

**Figure 7:**
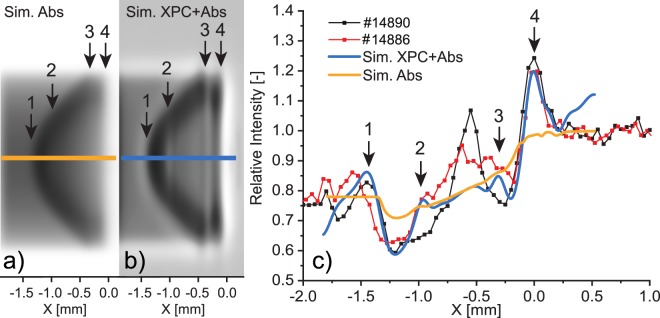
Simulated images of a shock-wave using the experimental parameters as input; (**a**) (*Sim.Abs*) shows only the absorption contribution, (**b**) (*Sim. XPC* + *Abs*) shows the XPC-effect in addition to the absorption contribution. The greys scales are different in the two images, the background (vacuum) has the same values in the two images. (**c**) Simulated profile along the object axis (blue and yellow line) superimposed to the experimental values (shots #14890, #14886). The scatter-point lines are calculated from a 10-pixel average along the object axis as indicated by the red box in Fig. 5. The black arrows (number 1–4) point toward features that are clearly visible in the experimental images and in the XPCI simulation but not in the absorption one.

In Fig. 5, we present some raw phase contrast images that clearly display the matter-vacuum and compressed-uncompressed matter interfaces in laser-driven shock-waves. Note that the pump laser was not perfectly aligned with the object on the last two shots (#14902, #14896). In Fig. 6, we show the same images but filtered with the so-called *“wrong filter”* (in *Method*). As it will be explained in the *Method* section, this filter allows reducing noise while enhancing the edge-contrast. We have added a dashed line to highlight the position of the shock front and used white arrows to indicate the laser interaction point. The shock front is clearly visible in all the images. In addition other, more subtle features can also be discerned. In shot #14902, for example, one can see a plastic shrapnel blown off during the interaction (see Fig. 6). In shot #14896 instead, the laser illuminated the metal support (white arrow), resulting in a shock-wave driven by the emitted radiation, which propagates parallel to the object axis. In Fig. 5, the images #14886 and #14890 show two perfectly-aligned shots. The images were obtained using identical laser pulse parameters: energy, temporal length and delay between the pump and the probe (8.3 ns). In shot #14886, however, the phase contrast is lower because the X-ray source intensity was 30% lower than for shot #14890, which means a lower signal-to-noise ratio. Moreover the source dimensions were larger on shot #14886 (10% and 20% larger along the *X-axis* and *Y-axis* respectively). The finite-source size acts as frequency filter, smoothing and removing the oscillations due to phase-contrast. In Fig. 7c, we compare the profiles taken over an area along the object axis (red box in Fig. 5) with the profiles from the simulated images in Fig. 7a,b. Figure 7a is generated taking in account only the absorption contribution, while in Fig. 7b both effects (absorption and phase-contrast) are taken in account. The *Sim. XPC* + *Abs*. simulation reproduces the object boundary (arrow 4 in Fig. 7), which is still intact 8.3 ns after the laser interaction. The shock front (arrow 1 in Fig. 7) is also well reproduced: the discrepancy of 13 μm between shot #14886 and the simulation is commensurate with the imaging resolution (10 μm) in the object plane. Instead, the XPCI code fails to reproduce structural features between the shock front and the object boundary (between arrows 2 and 3 in Fig. 7). This behaviour can be explained by the presence, of a strong density gradient behind the shock-front, feature not shown by the hydrodynamic simulations. In ref. ^18^ we argued that the discrepancy between the arrows 2 and 3 as a consequence of high intensity spikes inside the laser focal spot (no smoothing techniques were used). To mimic this behaviour we performed several hydrodynamic simulations where the central spike inside the laser spot was reduced from 50 m down to 5 m. In this way, we proved that a spike in laser intensity can effect the resulting XPC-image. The double border observable in Fig. 7b, indicated by the black arrows 3 and 4, is caused by the geometry of the imaging system. The source is not aligned with the object vertical face, producing a double-layer effect. This was also seen for shot #14890 in Figs. 5 and 6.

The simulated profile from the absorption image (*Sim. Abs* in Fig. 7c), does not reproduce all the features (arrows 1–4) observed in the experimental image. The object boundary (arrow 4) and the shock front (arrow 1) are not clearly identifiable in the absorption image.

Before one can extract quantitative information from a phase-contrast image, target homogeneity must also be taken into account. The phase-retrieval algorithm requires a homogeneous object, which means that the ratio *δ*/*β* should be constant over the entire object for all wavelengths. To prove that this assumption is justified, a simulated shock-wave was used as a test and the ratio *δ*/*β* was calculated across the object for all relevant wavelengths. We find that the requirement is fulfilled since emission lines from the source were far from the absorption edges of the elements composing our object^44^.

**Figure 8:**
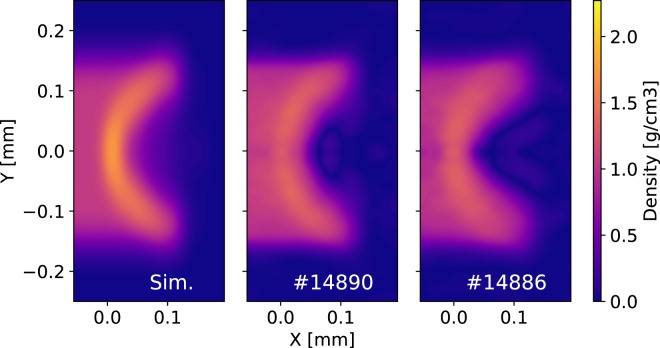
Density map of the object volume: Sim. simulated map blurred according to the measured source size; #14886 evaluated from image in Fig. 5 #14886; #14890 evaluated from image in Fig. 5 #14890.

In Fig. 8, we show the density map generated by the hydrodynamic code (blurred according to the source size measured in the experiment) on the left-hand side, and the two maps retrieved from experimental images (shots #14886 and #14890) on the right. The measured density maps are the result of a phase-retrieval code plus a Feldkamp-Davis-Kress (FDK) tomographic reconstruction algorithm^45^ was used. FDK is an algorithm suitable for point-projection tomography. A tomographic algorithm was chosen instead of Abel inversion for the following. First, the idea is to implement the diagnostic also at facilities where it is possible to acquire multiple projections at different angles. Second, one of the main problems of Abel inversion is its instability with noise fluctuations. The FDK algorithm is more stable because it works in the Fourier space, with a consequent smoothing of the noise fluctuation. Of course, an external Fourier filter could be applied before proceeding with an Abel inversion. The problem would be optimizing the filter magnitude in order to smooth noise without compromising the data.

**Figure 9:**
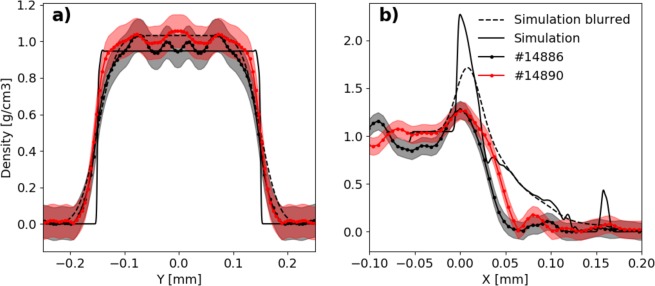
Density profiles of the maps showed in Fig. 8: the continuous lines represent simulated profiles, the scatter-lines are taken from experimental results and the dashed lines are the result of Gaussian smoothing of simulation data. The coloured areas (red and black) are the errors correspond to the experimental data.

The reconstructed slices along the object axis (#14886 and #14890 in Fig. 7) show the advancement of the shock-wave inside the unperturbed object. In Fig. 9a, we compare the density profile along the *Y-axis* (in front of the shock-front) with the theoretical prediction. We see that the experimental curve follows the theoretical one, noting that modulations on top of the experimental line are artefacts of the reconstruction code. In Fig. 9, dashed lines represent the density profile convolved with a Gaussian function in order to reproduce the imaging system resolution (the values used are 10.6 μm × 27 μm as estimated above). For the Y-profile, there is a good agreement between the experimental profile and the blurred simulated profile on both shots.

Figure 9b instead, shows a significant discrepancy between the simulated density profiles along the object axis and the reconstructed ones. Here, the hydrodynamic code estimates a shock front density of 2.3 g/cm^3^, while the density inferred from the experimental images is 1.3 g/cm^3^. The dashed line is the simulated profile which is treated with the same Gaussian filter as before. The shape of the shock is roughly reproduced by the simulation, however, the peak density of 1.7 g/cm^3^ is still higher than the measured value. Furthermore, the behaviour of the density behind the shock is quite different in the simulation and the experiment. The reconstructed density profiles show a faster decay than the simulated one behind the shock. This discrepancy can be explained by the difficulty of accurately modelling the laser-target alignment and the detailed intensity distribution of laser focal spot in the hydrodynamic simulator DUED^36^. It was not possible to acquire an image of the focal spot and pin down exactly its position on the object face during the experiment (especially along the horizontal axis). Every shot required to repeat the laser alignment and focusing procedure for both backlighter and object in order to compensate for the differences between each target.

## Discussion

The use of XPCI for shock-wave imaging and characterisation has been reported in several previous works^16,18–21^, which demonstrated how XPCI is superior to X-ray absorption imaging in shock detection. However, only recently has XPCI was demonstrated to work also with for broadband laser-plasma X-ray source^18,19^, in a configuration similar to the one employed in ICF facilities.

In this paper, we have presented an analysis of laser-driven shock-waves using a laser-plasma X-ray backlighter. XPCI allowed obtaining higher object definition than absorption radiography^6–8^. We verified that XPCI can reveal structural features that are undetectable using x-ray absorption techniques^3,5^ (see Fig. 7). Enhanced contrast at density interfaces, that occurs in XPCI, allows for clear identification of the shock front.

XPCI, as absorption radiography^6^, is also suitable for quantitative analysis of the acquired image. In this work, we have employed two approaches. In the first one we have tried to reproduce the experimental images starting from hydrodynamic simulations. In the second, we have used the acquired images to generate density maps and comparing them to the hydrodynamic simulations. Both ways have shown discrepancies between simulated and experimental data. As explained above, these discrepancies can be attributed to the difficulty of identifying the complex experimental parameters (e.g. laser alignment and laser spot morphology) that are used as inputs to the hydrodynamic simulations.

## Methodology

### Image simulation

The ideal intensity distribution of a XPC-image (the quantity *I*_*ideal*_ in Eq. 1) is given by ${I}_{0}\parallel \tilde{u}({P}_{1}){\parallel }^{2}$, where *I*_0_ is the incoming intensity on the object and $\tilde{u}({P}_{1})$ is the complex-valued wave-field distribution at the detector plane^32,34,37^:

2 $$\tilde{u}({P}_{1})=-\,\frac{i}{2\lambda }r{e}^{-ikr}{\iint }_{{{\mathbb{R}}}^{2}}[\frac{{R}_{0}}{{r}_{0}}+\frac{{R}_{1}}{{r}_{1}}]\frac{t(P){e}^{ik({r}_{0}+{r}_{1})}}{{r}_{0}{r}_{1}}\,dxdy$$

where *r* is the length of the vector from the source *S* to the point *P*_1_ on the detector plane, *t*(*P*) is the object transfer-matrix, *k* is the wave number, *R*_0_ and *R*_1_ are respectively the distance source-object and object-detector, *r*_0_ and *r*_1_ are the lengths of the vectors *SP* and *PP*_1_ respectively (see Fig. 1 for details). Equation 2 is sufficient to generate a phase-contrast image. Nevertheless, two considerations are necessary. First, the computation of a triple integral is required (*t*(*P*) is the result of an integral). To implement Eq. 2 in a code at least six loops (for a 2D image) have to be used, resulting in a large computational time. Second, here the source has been considered point-like. In a real experiment, the source has a finite size producing a blurring effect on the detected image.

However, if the waves propagate a long distance after the object and the transverse dimension of the sample itself is small compared to the propagation distance, the Fresnel approximation (or paraxial *R*_0_/*r*_0_ ≃ *R*_1_/*r*_1_ ≃ 1) can be used. This requires the inequality *N*_*eff*_ > 1 to be fulfilled where *N*_*F*,*eff*_ is the so-called effective Fresnel^4^ number equal to:

3 $${N}_{F,eff}={(R\bar{\lambda })}^{-1}\frac{M}{M-1}{\sigma }_{obj}\sqrt{{M}^{2}{\sigma }_{obj}^{2}+{(M-1)}^{2}{\sigma }_{src}^{2}+PS{F}_{\det }^{2}}$$

where *R* = *R*_1_ + *R*_0_, $\bar{\lambda }$ is the weighted sum of the source spectrum wavelength (Eq. 10), *M* = (*R*_0_ + *R*_1_)/*R*_0_ is the magnification, *σ*_*obj*_ is the width of the smallest object feature, *σ*_*src*_ is the source width and *PSF*_*det*_ is the detector PSF. Considering the parameters of the employed imaging system, an average photon energy around 7.5 keV, the average source and detector PSF ranging respectively between 5 ÷ 50 μm and 100 ÷ 200 μm, *N*_*eff*_ > 1 is fulfilled.

Equation 2 can then be reduced to a convolution integral and solved using Fourier transform. Applying the Fresnel approximation, Eq. 2 in the Fourier space becomes:

4 $$\tilde{U}(u,v)={M}^{2}T(Mu,Mv)\exp (\,-\,\pi i\lambda {R}_{1}M({u}^{2}+{v}^{2}))\exp \,[2\pi i\frac{{R}_{1}}{{R}_{0}}({x}_{0}u+{y}_{0}v)]$$

where *u* and *v* are the spatial frequencies corresponding to the variables *x*_1_ and *y*_1_, and *T*(*Mu*, *Mv*) is the transform of the object transfer function *t*(*P*). This last quantity is a function of (*Mu*, *Mv*) because the transform operator is applied with respect to *x*_1_. In this form, the equation is suitable for numerical evaluation using the Fast Fourier Transform (FFT) algorithm. The sample is described in the above formalism with the transfer function *t*(*P*):

5 $$t(P)=\exp [iD(P)-B(P)]$$

where the two quantities in the argument of the exponential are obtained from the following integrals:

6a $$D(P)=\frac{-2\pi }{\lambda }\int dr\delta (x,y,z);$$

6b $$B(P)=\frac{2\pi }{\lambda }\int dr\beta (x,y,z)$$

The two integrals are calculated along the path from the source point *S* to the detector point *P*_1_. Equations 5 and 6 are only valid in the thin sample approximation. A sample can be considered thin as long as the smallest features to be imagined are larger than the quantity $\sqrt{\lambda d}$, where *d* is the sample thickness^40^. At this level, the above equations are coupled with the hydrodynamic code. In particular, the two-dimensional density map generated by a hydrodynamic code in cylindrical geometry is used as an input to Eq. 6. In order to increase the calculation speed, the code uses a dedicated FORTRAN library, written for such a purpose, that performs all the calculations in parallel mode via OpenMP library. The transfer matrix is later used to generate the synthetic image considering all the parameters of the real imaging system, namely polychromaticity, source and detector *PSF*. Regarding the polychromaticity, for a polychromatic source the final image is a weighted sum of the monochromatic ones^32,40^:

7 $${I}_{poly}=\sum _{\lambda }\,w(E){I}_{mono}(\lambda )$$

### Image analysis

The condition required to use the Eq. 4 together with that of object homogeneity, and an X-ray photon energy below 50 keV allow phase retrieving from the measured image, by using the Transport Intensity Equation (TIE)^46,47^. In particular, the object project phase is:

8 $$\varphi (P)=-\,\frac{\bar{\delta }}{\bar{\mu }}\,\mathrm{ln}\,[{ {\mathcal F} }^{-1}\,[\frac{ {\mathcal F} ({I}_{rel})}{4{\pi }^{2}{R}_{eff}\frac{\bar{\delta }}{\bar{\mu }}[{u}^{2}+{v}^{2}]{M}^{2}+1}]]$$

where ${ {\mathcal F} }^{-1}$ and ${\mathcal F}$ are the inverse and direct Fourier transform and *R*_*eff*_ = *R*_1_/*M* is the effective propagation distance. Equation 8 has been implemented in a numerical code via FFT.

As explained before, as far as the inequality *N*_*F*,*eff*_ > 1 is fulfilled, the above equations can also be applied in the case of a polychromatic source^48^. The quantities $\bar{\delta }$, $\bar{\mu }$, $\bar{\lambda }$ in Eq. 8 are then averaged over the source spectrum and the detector function response *D*(*λ*):

9 $$\bar{\mu },\bar{\delta }=\frac{\int \mu (\lambda ),\delta (\lambda )D(\lambda )I(\lambda )d\lambda }{\int D(\lambda )I(\lambda )d\lambda }$$

10 $$\bar{\lambda }=\frac{\int \lambda D(\lambda )I(\lambda )d\lambda }{\int D(\lambda )I(\lambda )d\lambda }$$

Prior to proceeding with phase retrieval, the image is processed with a Wiener filter^49^ in the Fourier space:

11 $$W(u,v)=\frac{PS{F}^{\ast }}{|PSF{|}^{2}+K}$$

where *PSF* is the total point spread function (*PSF*_src_*PSF_*det*_) and *K* is a constant that takes noise variance in account. The filter has two effects: the first one is to partially remove blurring induced by the imaging system and the second is to normalize the noise.

One consideration can be done on Eq. 8. The term 4*π*^2^ on the denominator is required by the FFT used for the numerical evaluation of *I*_*rel*_. Removing such term from the filter will result in a non-physical behaviour at the object border, and in general at all the interfaces. The result is an enhancement of the contrast at the border. We found useful applying such “wrong filter” on the measured images for qualitative analysis.

The calculated projected phase map can be used to evaluate a three-dimensional map. Since only one projection is available, this requires that the object is cylindrical. The reconstruction is performed with a Feldkamp-Davis-Kress (FDK) algorithm^45,50,51^. It allows performing a tomographic reconstruction starting from a set of cone-beam projections of the object. In our case, the phase-map projection *φ*(*P*) at the object plane, obtained from Eq. 8, is used to generate a three-dimensional spectral averaged^50,52^:

12 $$\varphi {({\bf{x}})}_{poly}=-\,\frac{{R}_{0}^{2}d}{2}{\int }_{0}^{2\pi }\,\frac{1}{{p}^{2}}{ {\mathcal F} }_{1}^{-1}\,[|Mv|{ {\mathcal F} }_{1}\,[\frac{\varphi (P,\alpha )}{\sqrt{{R}_{0}^{2}+|P{|}^{2}}}]]\,d\alpha $$

where ***x*** = (*x*, *y*, *z*) is a vector describing the object volume, *d* is the distance *R*_0_ minus the radius of the cylinder containing the object, and

13 $$p=d-x\,\cos (\alpha )-y\,\sin (\alpha )$$

The three-dimensional distribution of $\bar{\delta }({\bf{x}})=\varphi {({\bf{x}})}_{poly}/k$ can be reconstructed from the phase, and finally the density distribution is obtained solving the equations

14 $$\delta =\frac{{r}_{e}{N}_{A}{\lambda }^{2}\rho }{2\pi }\sum _{j}\,\frac{{w}_{j}[{Z}_{j}+{f}_{j}^{^{\prime} }]}{{A}_{j}}$$

where *r*_*e*_ is the classical electron radius, *N*_*A*_ is the Avogadro number, *ρ* is the mass density of the compound, *Z*_*j*_ and *A*_*j*_ are the atomic number and the atomic weight of *j*th element of the compound, ${f}_{j}^{^{\prime} }$ is the real part of the dispersion correction factor.

The parameters in Eq. 14 are evaluated using the library *Xray-lib* described in ref. ^53^.
