# Author Correction: Quantitative phase contrast imaging of a shock-wave with a laser-plasma based X-ray source

**DOI:** 10.1038/s41598-020-65141-7

**Published:** 2020-05-12

**Authors:** F. Barbato, S. Atzeni, D. Batani, D. Bleiner, G. Boutoux, C. Brabetz, P. Bradford, D. Mancelli, P. Neumayer, A. Schiavi, J. Trela, L. Volpe, G. Zeraouli, N. Woolsey, L. Antonelli

**Affiliations:** 1 Empa, Materials Science and Technology, 8600 Dübendorf, Switzerland; 2 Dipartimento SBAI, Università di Roma “La Sapienza”, 00161 Rome, Italy; 3 Universitè de Bordeaux, CNRS, CEA, CELIA, UMR 5107, F-33405 Talence, France; 4 GSI Helmholtzzentrum für Schwerionenforschung GmbH, 64291 Darmstadt, Germany; 5 Donostia International Physics Center (DIPC), 20018 Donostia, Spain; 6 CLPU, Centro de Laseres Pulsados, Building M5, 37185 Villamayor, Salamanca Spain; 7 Universidad de Salamanca, Patio de Escuelas 1, 37008 Salamanca, Spain; 8 Department of Physics, York Plasma Institute, University of York, York, YO10 5DD United Kingdom; 9 National Research Nuclear University MEPhI, Department of Plasma Physics, 115409 Moscow, Russia

Correction to: *Scientific Reports* 10.1038/s41598-019-55074-1, published online 11 December 2019

This Article contains a repeated typographical error where the unit “µm” is incorrectly given as “m” or “um” and “°” is incorrectly given as “deg”.

